# Echocardiographic parameters in patients with and without hypotension during dialysis

**DOI:** 10.34172/jcvtr.2021.41

**Published:** 2021-08-25

**Authors:** Hoorak Poorzand, Farzaneh Sharifipour, Abdollah Kerachian, Fereshteh Ghaderi, Hedieh Alimi, Leila Bigdelu, Afsoon Fazlinejad, Negar Morovatdar, Banafsheh Ataei

**Affiliations:** ^1^Vascular and Endovascular Surgery Research Center, Faculty of Medicine, Mashhad University of Medical Sciences, Mashhad, Iran; ^2^Kidney Transplantation Research Center, Department of Internal Medicine, Mashhad University of Medical Sciences, Mashhad, Iran; ^3^Faculty of Medicine, Mashhad University of Medical Sciences, Mashhad, Iran; ^4^Department of Cardiology, Ghaem Hospital, Mashhad University of Medical Sciences, Mashhad, Iran; ^5^Clinical Research Unit, Faculty of Medicine,Mashhad University of Medical Sciences, Mashhad, Iran

**Keywords:** Intradialytic Hypotension, Transthoracic Echocardiography, Left Ventricular Ejection Fraction, Global Longitudinal Strain

## Abstract

***Introduction:*** Hypotension during dialysis is a common complication of hemodialysis and is associated with increased patient mortality and morbidity. Intradialytic hypotension is a decrease in systolic BP ≥20 mm Hg or a reduction in mean arterial pressure by 10 mm Hg along with clinical events and the need for correction. This study compares cardiac function, using transthoracic echocardiography with strain modality in patients with intradialytic hypotension with those without hypotension during dialysis.

***Methods:*** We studied 60 patients with chronic renal failure undergoing regular hemodialysis from April 2018 to February 2019. We compared thirty patients in the intradialytic hypotension group, with the remaining 30 patients in the control group. We did transthoracic echocardiography a day after hemodialysis using conventional, tissue doppler, and strain imaging.

***Results:*** Early diastolic mitral annulus velocity (e’) was lower in the intradialytic hypotension group in comparison with the control group which their difference was statistically significant (5.540 ± 1.51 versus 6.920 ± 1.98, *P* value:0.007) Left Ventricular Ejection Fraction (LVEF) was also significantly lower in the intradialytic hypotension group (51.07 ± 8.714 versus 59.43 ± 4.133, *P* value < 0.001). Global Longitudinal Strain (GLS) was significantly lower in the intradialytic hypotension group (-14.17 ± 2.79 versus -18.99 ± 2.25, *P* value < 0.001). The receiver operator characteristics (ROC) curve point-coordinates that GLS of -16.85 and lower (more positive) has 83% sensitivity and 87% specificity for intradialytic hypotension.

***Conclusion:*** The echocardiographic assessment could be used as a tool for the prediction of hypotension during dialysis.

## Introduction

Intradialytic hypotension (IDH) is a familiar complication of hemodialysis, which increases mortality and morbidity in patients.^1^ Conforming to the Kidney Disease Outcomes Quality Initiative (K/DOQI) guidelines, intradialsis hypotension is the reduction in systolic blood pressure ≥20 mm Hg or a decrease in mean arterial pressure by 10 mm Hg along with clinical incident and needs for treatment.^2^

When plasma volume decreases during ultrafiltration, vascular resistance and cardiac output increase to maintain blood pressure. IDH occurs when these mechanisms do not work correctly.^3^ The decreased sympathetic tone in some patients could reduce arteriolar resistance, increase blood pooling in veins, and ultimately reduce blood pressure.^4^

A decline in blood volume and insufficient compensatory mechanism may cause the underfilling of the heart and activation of Bezold–Jarish reflex and ultimately sudden hypotension.^5^ Since diastolic dysfunction and LVH reduce ventricular filling and stroke volume, they can contribute to intradialytic hypotension.^6,7^

Cardiac disease, including systolic or diastolic function impairement, augments the risk for intradialytic hypotension.^8^

Demographic risk factors for intradialysis hypotension include older age, diabetes mellitus, longer dialysis vintage, lower pre-dialysis blood pressure, female gender, and higher body mass index.^1^

Consequences of intradialytic hypotension can range from cramping and post-dialysis fatigue to bowel ischemia, cardiovascular events, stroke, and access thrombosis. Intradialytic hypotension may reduce our ability to provide adequate dialysis when stopped in the early stage at the patient’s request.^9^

Tatsuya Shoji et al suggested that orthostatic hypotension after hemodialysis and intradialytic hypotension are independent factors, increasing hemodialysis patients’ mortality.^10^

Speckle Tracking Echocardiography (STE) could reveal any alteration in systolic function.^11^

Among those cases of chronic renal failure with normal left ventricule systolic function, 2-dimensional speckle tracking is a growing modality to identify the subtle change of a cardiac function.^12,13^

The present study evaluates whether patients with intradialytic hypotension and patients without hypotension during dialysis differ in the cardiac function assessed by transthoracic echocardiography with strain modality.

## Materials and Methods

### Patients

This cross-sectional study was conducted in Imam Reza and Ghaem Hospitals in Mashhad from April 2018 to February 2019. All participants signed written informed consent.

This study included 60 patients with chronic kidney disease on regular hemodialysis. Hemodialysis was done three times per week with high flux. All patients have KT/V > 1.1.

Exclusion criteria included acute renal failure, acute coronary syndrome, cerebral ischemic or hemorrhagic infarct within three months, conduction disturbance, pericardial disease, at least moderate valvular heart disease, malignancy, chronic obstructive pulmonary disease (COPD), any uncontrolled metabolic or infectious disease, and low echocardiographic image quality.

Thirty patients were in the intradialytic hypotension group, and the remaining 30 patients were in the control group without hypotension during hemodialysis.

The method of sampling in our study was a convenience method based on inclusion criteria.

Blood pressure is recorded every 30 minutes in the supine position during hemodialysis.

Intradialytic hypotension is a reduction in systolic BP ≥20 mm Hg or a reduction in mean arterial pressure by 10 mm Hg, along with clinical incidents and the necessity for correction.

### Conventional echocardiography

Transthoracic echocardiography was done before hemodialysis, using Siemens ACUSON SC2000 Ultrasound System with 4v1c Transducer (Frequency Bandwidth: 1.25 – 4.5 MHz).

We evaluated Left ventricle end diastolic volume index (LVEDV index;cc/m^2^),Left atrium volume index (LAVI; cc/m^2^), mid diameter of right ventricle (mid RV; cm), systolic pulmonary artery pressure (PAP; mm Hg), early diastolic mitral inflow velocity(E ;cm/sec), late diastolic mitral inflow velocity(A;cm/sec), early diastolic mitral annulus velocity (e’velocity; cm/sec), Mitral E/e’ ratio, left Ventricle ejection fraction (LVEF;%) and left ventricule global longitudinal strain (GLS ;%).

We measured LV end-diastolic and systolic volume index, LVEF (by biplane Simpson’s method), and right ventricular size (using the mid-diameter of RV in RV-focused view) based on the latest guideline of American Society of Echocardiography (ASE).^14^

LA volume index was measured by using a biplane disk summation technique. The upper limit of normal for the LA volume index is 34 mL/m^2^in both genders.^14^

For LV diastolic function, peak E and A-wave velocities, e’ velocity, and E/e’ ratio were assessed according to a recommendation of ASE.^15,16^

### Velocity Vector Imaging (VVI)

Strain,as a myocardial deformation index,is an echocardiographic parameter measured throughout the cardiac cycle, using standard B-mode images for speckle tracking analysis(STE).^17,18^

In 2-dimensional STE, Global Longitudinal Strain (GLS) is the ratio of the maximal longitudinal systolic change in the length of myocardium into its initial size. In systolic phase, the longitudinal length of the myocardium decrease,resulting in GLS defined as negative values, with more negative values meaning as better LV systolic function.^19^

VVI is a new echocardiographic modality, merging speckle tracking with endocardial border detection. VVI is also angle independent like STE, but seems to be simpler with more rapid tracking and processing times. ^17,20^ It needs just a single frame tracing of the endocardial border for extracting the quantitative data.^21^

**Figure 1:**
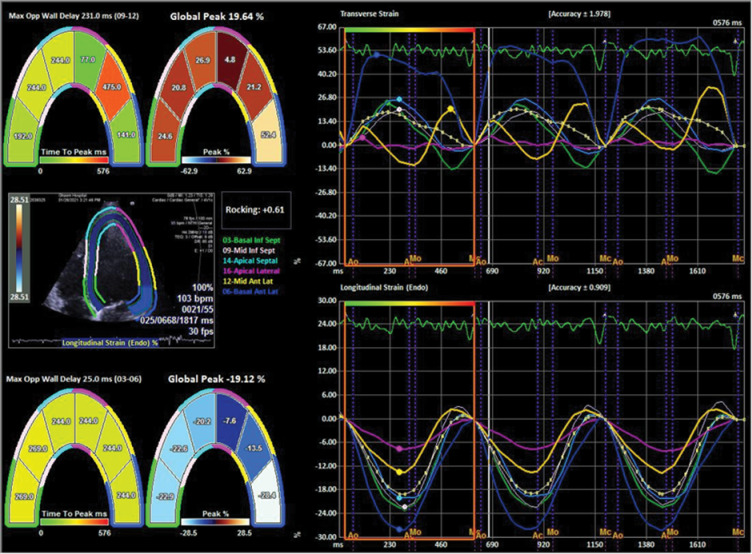
Strain analysis of left ventricle for evaluation of global longitudinal strain(GLS) using velocity vector imaging.

Three apical views (apical four-chamber, two-chamber, and long-axis views), are needed for VVI analysis of LV, with GLS calculated as the average of strain in all myocardial segments. Figure 1 shows a strain analysis of LV for evaluation of GLS. Measurements begin with the apical long-axis view to define the time of aortic valve closure.^14^

Pulmonary artery pressure was measured using tricuspid regurgitation doppler signal.

### Statistics

Statistical analysis was done by Statistical Package for Social Sciences (SPSS) software, release 22.0.0 for Windows (IBM Corporation). (SPSS, Inc. - Chicago, IL)

Categorical variables were described as frequency and percentage. Quantitative variables were defined as the mean  ±  standard deviation. The chi-square test was used to analyze categorical variables: Independent-Samples Student’s t-test and Mann–Whitney test used to compare parametric and non-parametric data, respectively. The receiver operator characteristics (ROC) curve was used to estimate a cut-off value for GLS as a predictor for intradialytic hypotension. A *P* value < 0.05 considered to be statistically significant.

## Results

Thirty patients were in the intradialytic hypotension group, and 30 patients were in the control group (Table 1).

**Table 1:** Frequency and comparison of demographic data between groups

**Baseline data**	**Intradialysis blood pressure**		***P*** ** value**
	**With hypotension(No#30)**	**Without hypotension(No#30)**	
Age (mean ± SD)	56.03 ± 15.35	52.57 ± 14.25	0.293
Male*	14 (46.67%)	18 (60%)	0.301
BMI (mean ± SD)	25.45 ± 5.76	24.95 ± 3.95	0.817
Diabetic*	11 (36.67%)	11 (36.67%)	> 0.99
Hypertensive*	18 (60%)	12 (40%)	0.602
CAD*	6 (20%)	3 (10%)	0.47

Abbreviation: BMI, Body mass index ; CAD (coronary artery disease) is defined as a history of CABGs, PCI, or RWMA in echocardiography. A *P*-value < 0.05 considered to be statistically significant. * data presented as number (percentile)

There were 16 females and 14 males in the intradialytic hypotension group and 12 females and 18 males in the control group; their difference was not statistically significant (*P* value = 0.301).

The number of diabetic cases in the intradialytic hypotension group was equal to the control group (*P* value = 1.00).

Eighteen patients in the intradialytic hypotension group were hypertensive compared with 16 patients in the control group; their difference was not statistically significant (*P* value: 0.602).

Six patients in the intradialytic hypotension group had coronary artery disease compared to three patients in the control group, whose difference was not statistically significant (*P* value: 0.47). Our study’s coronary artery disease is defined as a history of CABGs, PCI, or RWMA in echocardiography.

There was no statistically significant difference between the two groups concerning age (56.03  ±  15.35years versus 52.57  ±  14.25years, *P* value: 0.293), BMI (25.45  ±  5.76 versus 24.95  ±  3.95, *P* value = 0.817), LVEDV index, LAVI, mid RV, PAP, E and A wave velocity and E/E_m_ (Table 2).

**Table 2:** Comparision between echocardiographic parameters in intradialytic hypotension group and control group

**ECHO Parameter**	**Measurements in intradialytic hypotension group**	**Measurement in the control group (without intradialytic hypotension)**	***P*** ** value**
LVEDV index, mL/m^2^	62.73 ± 16.271	63.40 ± 12.045	0.722
LA VI, mL/^m^2	36.03 ± 9.463	37.87 ± 13.881	0.767
LVEF (%)	51.07 ± 8.714	59.43 ± 4.133	< 0.001
LV GLS (%)	-14.17 ± 2.79	-18.99 ± 2.25	< 0.001
Mid RV (cm)	2.973 ± 0.25	3.023 ± 0.39	0.693
Early diastolic mitral inflow velocity(E): cm/s	76 ± 24.065	85.27 ± 25.187	0.229
Late diastolic mitral inflow velocity(A):cm/s	86.17 ± 19.580	79.73 ± 21.846	0.222
Early diastolic mitral annulus velocity (e’):cm/s	5.540 ± 1.51	6.920 ± 1.98	0.007
Mitral E/e’	13.957 ± 4.45	12.987 ± 4.19	0.563
PAP, mm Hg	31.27 ± 9.373	33.53 ± 9.092	0.365

Abbreviation: LVEDV, left ventricular end-diastolic volume; LAVI, left atrial volume index; Mid RV, mid portion diameter of the right ventricle; PAP, systolic pulmonary artery pressure; LVEF, left ventricular ejection fraction; GLS, left ventricular Global longitudinal strain. All echocardiographic parameters were expressed as mean  ±  SD. A *P* value < 0.05 considered to be statistically significant.

LAVI was larger than normal in both groups (> 34 mL/m^2^), but there was no significant difference between the two groups (36.03 ±  9.463 versus 37.87  ±  13.881, *P* value = 0.767).

Early diastolic mitral annulus velocity (e’) was lower in the intradialytic hypotension group in comparison with the control group which their difference was statistically significant (5.540 ±  1.51 versus 6.920  ±  1.98, *P* value:0.007), LVEF was also significantly lower in the intradialytic hypotension group (51.07  ± 8.714 versus 59.43  ±  4.133, *P* value < 0.001).

**Figure 2:**
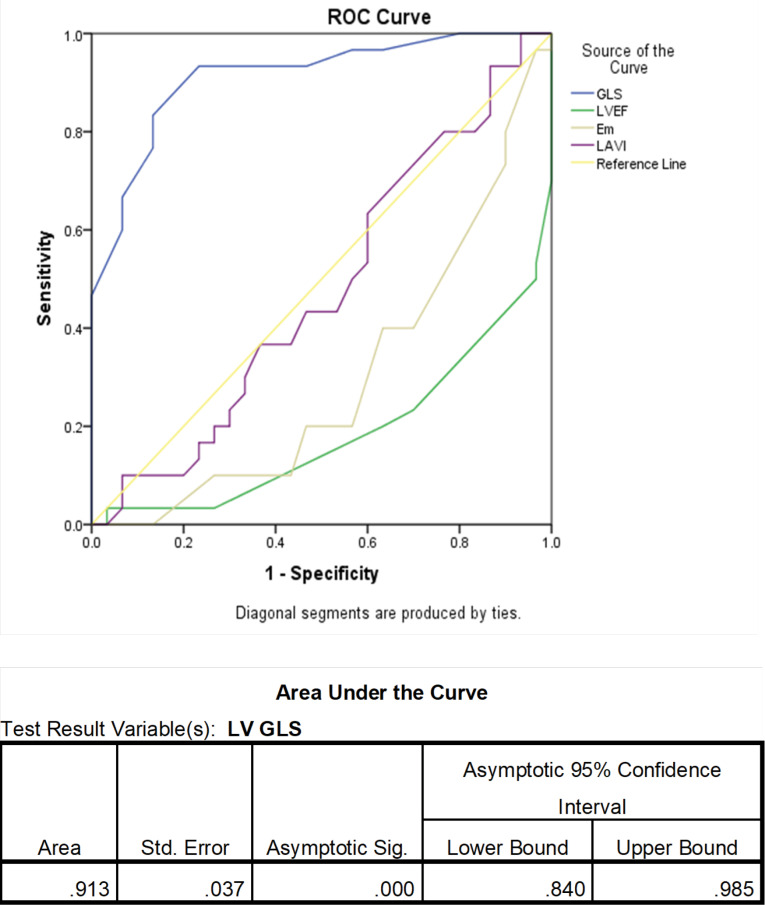
ROC curve analysis for left ventricle global longitudinal strain

GLS was significantly lower in the intradialytic hypotension group (-14.17  ±  2.79 versus -18.99  ± 2.25, *P* value < 0.001), and by using the ROC curve, we also figure out that GLS of -16.85 and lower (more positive) could predict intradialytic hypotension (sensitivity:83%, specificity: 87%) Figure 2 shows ROC curve analysis for LV GLS. AUCs (area under the curve) in LVEF, e’, and LAVI were 0.18, 0.29, and 0.47, respectively.

## Discussion

In this study, transthoracic echocardiography using strain indices was applied to evaluate the echocardiographic data in patients with and without intradialytic hypotension. We concluded that LVEF, as an indicator of systolic function and GLS were significantly lower in hypotension-prone hemodialysis patients. E’ velocity as a marker of diastolic function was significantly lower in patients with hypotension during dialysis. We also configure that GLS can be used as a parameter to predict intradialytic hypotension. GLS of -16.85 and lower (more positive) could predict intradialytic hypotension (sensitivity:83%, specificity: 87%).

Intradialytic hypotension is common. The replacement the intravenous fluid is needed to enable patients to complete the dialysis session. Intradialytic hypotension decrease the duration and efficacy of the dialysis and increases the adverse effects associated with hemodialysis. ^22^

Myocardial contractility may increase to compensate for blood volume depletion, so in patients with impairement of systolic function, the blood pressure decrease might be more evident.^8^

The relationship between survival and blood pressure in dialysis patients has remained controversial. Hypertension has an essential role in developing cardiovascular complications, the main cause of mortality and morbidity in hemodialysis cases.^10^

Kramann, et al used STE for early detection of uremic cardiomyopathy and to predict cardiovascular mortality in dialytic patients. They showed that LV GLS was independently associated with all-cause mortality.^23^

Krishnasamy,et al similarly defined that GLS was independently related to all-cause mortality in the stage 4 and 5 chronic kidney disease.^24^ In another study by Hensen, et al on pre-dialysis and dialysis patients, LV GLS (*P* <  0.041) and LV ejection fraction (*P* <  0.019) were related with all-cause mortality, and renal transplantation was less frequently done in those with left ventricular GLS < 10.6%. ^25^

Poldermans D et al used dobutamine stress echocardiography to assess the inotropic reserve of left ventricle and also the presence of ischemia in the myocardium in patients with and without hypotension during dialysis. The baseline cardiac index was similar in the two groups. However, as cardiac index increases with dobutamine, the cardiac reserve was lower among patients prone to hypotension during dialysis and such abnormal findings,which were more common than the evidence of myoardial ischemia, assumed to have an important role in the hypotension during dialysis.^26^ In another study by Kolb J et al studied the relationship between cardiac function and intradialytic morbid events such as intradialytic hypotension and muscle cramps. They demonstrated that a high total peripheral resistant index and low cardiac index, detected in in the first 30 minutes of hemodialysis, could increase the risk of intradialytic morbid events.^27^

It should be added that the strain imaging could be helpful in detecting abnormal left ventricle function in hemodialysis patients, even without apparent LV systolic dysfunction.^28^

Diabetes mellitus (DM) and arterial hypertension (HTN) might alter GLS parameters, and such patients were not excluded from the study. However, a separate analysis was done, which showed no significant difference between the frequency of DM and HTN between the two study groups (*P*> 0.05 for both HTN and DM).

## Conclusion

Echocardiography using strain indices could play an important role in the dialytic patients for detecting subtle systolic and diastolic LV dysfunction and predicting intradialytic adverse events such as intradialytic hypotension.

## Acknowledgments

The authors would like to acknowledge the Clinical Research Development Unit, Imam Reza Hospital, Mashhad University of Medical Sciences.

## Competing interest

The authors have none to declare.

## Ethical approval

The local scientific committee of ethics approved this study (IR.MUMS.MEDICAL.REC.1397.065).

## Funding

None.
